# Superior Catalytic Selectivity by Merging Carbon-Decorated Pd Species with Zeolite Micropores

**DOI:** 10.1021/acscentsci.5c01175

**Published:** 2025-07-19

**Authors:** Hai Wang, Feng-Shou Xiao

**Affiliations:** † Key Lab of Biomass Chemical Engineering of Ministry of Education, College of Chemical and Biological Engineering, 12377 Zhejiang University, Hangzhou 310058, China

## Abstract

Carbon-decorated Pd species were encapsulated into siliceous silicalite-1 (S-1) zeolite, showing high activity and superior selectivity in hydrogenation of furfural to furfuryl alcohol.

## Graphical abstract

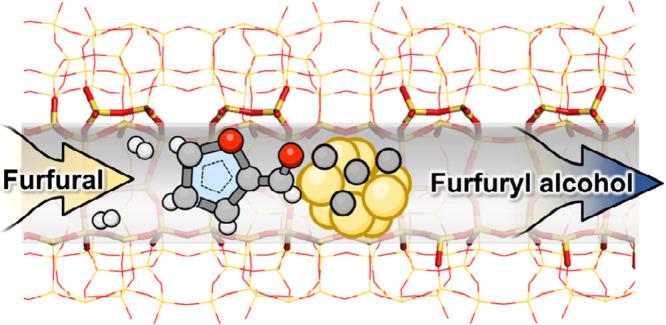

Zeolites are a class of inorganic crystalline materials with ordered micropores and excellent stability, which have been widely applied in petroleum refining and petrochemical industries.^1^ With the fast development of zeolite synthetic protocols,^2^ merging zeolite with active metal species has extended the applications of zeolite-based materials beyond the petrochemical industry to include many sustainable processes, such as fine-chemical synthesis and pollutant removal.^1^ Incorporation of active metal species into zeolite is an attractive method for synthesizing highly efficient heterogeneous catalysts, where the zeolite can not only stabilize the metal species against sintering but also enhance product selectivity.^3^ For example, Corma et al. showed that the localization of subnanometric Pt and PtSn clusters in the sinusoidal channels of S-1 zeolite provided good stability in propane dehydrogenation.^4^ We found that Pd nanoparticles (NPs) encapsulated in Beta-zeolite exhibited high selectivity in nitroarene hydrogenation due to the sterically selective adsorption of nitroarenes on the Pd NPs.^5^ These results demonstrate the importance of metal–zeolite synergism for boosting catalysis. In this issue of *ACS Central Science*, Yu and co-workers reported a Pt-C@S-1 catalyst with carbon-decorated subnanometric Pd NPs in S-1 zeolite,^6^ showing both high activity and superior selectivity in hydrogenation of furfural to furfuryl alcohol, an important sustainable process for conversion of biomass into value-added chemicals. Mechanistic studies reveal that the decoration of carbon on Pd species in micropores of S-1 zeolite significantly adjusts the adsorption of furfural in a linear mode (Figure 1a),^6^ which can then be hydrogenated into furfuryl alcohol. In contrast, flat-lying adsorption of furfural on the Pd NPs in S-1 zeolite leads to its hydrogenation to tetrahedrofurfuryl alcohol (Figure 1b). On the conventionally supported Pd NPs, furfural hydrogenation was not selective (Figure 1c).^7^

**Figure 1:**
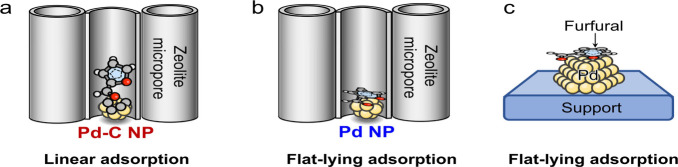
Scheme showing (a) linear adsorption of furfural on the Pd-C@S-1 catalyst and (b,c) flat-lying adsorption of furfural on the (b) Pd@S-1 and (c) conventionally supported Pd NP catalysts.

The Pd-C@S-1 catalyst was synthesized from a one-pot hydrothermal crystallization combined with a direct carbonization–reduction protocol, where the carbon-decorated Pd NPs, with an average size of about 0.9 nm, were successfully encapsulated into S-1 zeolite. In contrast, the carbon-free Pd@S-1 catalyst produced Pd NPs with an average size of 2.6 nm. Pd *K*-edge XANES spectra of the Pd-C@S-1 and Pd@S-1 showed that the Pd NPs were positively charged in both catalysts, which might be due to the interaction between Pd and oxygen atoms within the zeolite framework.

The catalytic performances of the Pd-C@S-1 and Pd@S-1 catalysts were evaluated in selective hydrogenation of furfural to furfuryl alcohol—where side reactions such as hydrogenolysis, decarbonylation, and ring hydrogenation generally occur. Under the employed reaction conditions (80 °C, 5 bar of H_2_, and water as solvent), the Pd-C@S-1 exhibited excellent performance, giving a 98% yield of furfuryl alcohol after a 90 min reaction. By contrast, the Pd@S-1 catalyst showed a 11.9% yield of furfuryl alcohol due to the low furfural conversion and over hydrogenation of furfuryl alcohol to tetrahedrofurfuryl alcohol. These data provide evidence of the high activity and superior selectivity for furfuryl alcohol over the Pd-C@S-1 catalyst.

Mechanistic investigations showed that the positively charged Pd species in the Pd-C@S-1 were favorable for H_2_ activation and furfural adsorption, thus enhancing the hydrogenation activity.

Owing to the presence of carbon-decorated Pd species in zeolite micropores, a linear adsorption of furfural on the Pd NPs in the Pd-C@S-1 existed, where the zeolite micropores efficiently stabilized this adsorption configuration.

This linear adsorption of furfural results in the excellent selectivity of furfuryl alcohol through hydrogenation. In contrast, the flat-lying adsorption of the furan rings in furfural on the Pd@S-1, due to the lack of carbon decoration, led to hydrogenation of the furan rings.

This work sheds light on a new approach to enhance the catalytic selectivity by merging carbon-decorated Pd species with zeolite micropores.

The carbon decoration not only modified the electronic structure of Pd species but also modulated the adsorption configurations of reactants in the zeolite micropores. It is anticipated that this brand-new strategy will be extended to other important reactions, especially those that require precise control of the molecular adsorption configurations, such as substituted nitroarenes hydrogenation,^5^ acetylene hydrogenation,^8^ and CO_2_ hydrogenation.^9^ Meanwhile, further research is needed to reach a 100% yield of furfuryl alcohol in order to decrease the cost of product separation and purification. In this regard, the wettability, morphology, or defects of the zeolite might be adjusted. For example, by localizing Pd NPs in S-1 zeolite with appropriate hydrophilicity (Pd@S-1-OH), a >99.9% yield of furan was obtained in furfural hydrogenation due to the fast desorption of furan in the hydrophilic zeolite micropores.^10^ Furfuryl alcohol selectivity might be enhanced by fine control of the molecular adsorption configuration on metal and diffusion in zeolite micropores. We expect that this work will inspire future studies on metal–zeolite catalyst design and application in the future.
